# Draft Genome Sequence of Desulfobacter hydrogenophilus DSM 3380, a Psychrotolerant Sulfate-Reducing Bacterium

**DOI:** 10.1128/MRA.00203-20

**Published:** 2020-04-02

**Authors:** Emma Bertran, Lewis M. Ward, David T. Johnston

**Affiliations:** a Department of Earth and Planetary Sciences, Harvard University, Cambridge, Massachusetts, USA; b Department of Geosciences, Princeton University, Princeton, New Jersey, USA; c Earth-Life Science Institute, Tokyo Institute of Technology, Tokyo, Japan; University of Southern California

## Abstract

Here, we describe the genome of Desulfobacter hydrogenophilus DSM 3380, a bacterium that belongs to the *Desulfobacterales*. The genome of this strictly anaerobic bacterium capable of sulfate reduction expands our understanding of microbial sulfate reduction in a wide range of environmental conditions.

## ANNOUNCEMENT

Desulfobacter hydrogenophilus DSM 3380 is a bacterium that was first described and isolated from enrichment culture taken from Rio di San Giacomo in Venice, Italy, by Friedrich Widdel (1). It belongs to the *Desulfobacterales* order within the bacterial domain and grows most optimally anaerobically at 30°C and pH 7.1 to 7.4 (1). However, *D. hydrogenophilus* can also thrive in the cold subseafloor and can perform microbial sulfate reduction at low temperatures (2). It is also reported to be capable of growth at a wide pH range of 5.5 to 7.6 (1). While *D. hydrogenophilus* is incapable of growth as a sulfur disproportionator, it is closely related to Desulfobacter curvatus, which has been shown to grow via disproportionation of sulfite or thiosulfate (2). The genetic capacities for sulfate reduction and sulfur disproportionation are not readily distinguishable from genome content alone (3). Therefore, this *D. hydrogenophilus* strain was sequenced as part of a larger effort to identify molecular markers for sulfur disproportionation via comparative genomic analysis of closely related sulfate reducers and sulfur disproportionators (4–6).

Purified genomic DNA of *D. hydrogenophilus* was acquired from the DSMZ. *D. hydrogenophilus* was grown anaerobically at 30°C in medium 195, and DNA extraction was performed using a JetFlex genomic DNA purification kit from Genomed. DNA libraries were produced with a Nextera XT library prep kit on a Hamilton Microlab Star automated liquid-handling system and then sequenced on an Illumina HiSeq system using the 250-bp paired-end protocol. Reads were adapter trimmed with Trimmomatic v0.30 (7). *De novo* assembly was performed using SPAdes v3.7 (8). Annotation was performed using RAST v2.0 (9). The publicly available genome was annotated with PGAP (10). CheckM v1.0.12 (11) was used to estimate genome completeness. MetaPOAP v1.0 (12) was used to determine the likelihood of the presence or absence of metabolic pathways. Taxonomic assignment of the genome was verified with GTDB-Tk v0.3.2 (13). Hydrogenase proteins were classified with HydDB (14). Default parameters were used for all software.

The *D. hydrogenophilus* genome was recovered at 272× coverage as 3,735,174 reads, which assembled into 457 contigs with an *N*_50_ value of 81,008 bp and a total of 5,105,199 bp encoding 5,458 coding sequences and 57 RNAs. The genome has a 47.0% GC content. CheckM estimates the genome to be 100% complete with 1.67% redundancy, 20% of which is due to strain heterogeneity.

The genome of *D. hydrogenophilus* encoded all genes required for microbial sulfate reduction. These include sulfate adenylyltransferase, adenylylsulfate reductase (subunits A and B), dissimilatory sulfite reductase (subunits A, B, and C), and the sulfite reduction-associated DsrMKJOP complex. In addition, genes encoding thioredoxin disulfite reductase, which is essential for the formation of intracellular reduced disulfide bonds in cells, were found. The *D. hydrogenophilus* genome also includes a group IV NiFe hydrogenase for H_2_ uptake and a molybdenum nitrogenase for N_2_ fixation.

### Data availability.

This whole-genome shotgun project has been deposited at DDBJ/ENA/GenBank under the accession number JAAGRP00000000. The FASTQ files of the raw reads were deposited in the NCBI SRA under accession number SRR11035949.
